# The role of conserved charged residues in the bidirectional rotation of the bacterial flagellar motor

**DOI:** 10.1002/mbo3.587

**Published:** 2018-03-24

**Authors:** Yasuhiro Onoue, Norihiro Takekawa, Tatsuro Nishikino, Seiji Kojima, Michio Homma

**Affiliations:** ^1^ Division of Biological Science Graduate School of Science Nagoya University Nagoya Japan

**Keywords:** bacterial motility, electrostatic interaction, flagellar motor, rotor–stator interaction, *Vibrio*

## Abstract

Many bacteria rotate their flagella both counterclockwise (CCW) and clockwise (CW) to achieve swimming toward attractants or away from repellents. Highly conserved charged residues are important for that motility, which suggests that electrostatic interactions are crucial for the rotor–stator function. It remains unclear if those residues contribute equally to rotation in the CCW and CW directions. To address this uncertainty, in this study, we expressed chimeric rotors and stators from *Vibrio alginolyticus* and *Escherichia coli* in *E. coli*, and measured the rotational speed of each motor in both directions using a tethered‐cell assay. In wild‐type cells, the rotational speeds in both directions were equal, as demonstrated previously. Some charge‐neutralizing residue replacements in the stator decreased the rotational speed in both directions to the same extent. However, mutations in two charged residues in the rotor decreased the rotational speed only in the CCW direction. Subsequent analysis and previous results suggest that these amino acid residues are involved in supporting the conformation of the rotor, which is important for proper torque generation in the CCW direction.

## 1 INTRODUCTION

**Figure 1:**
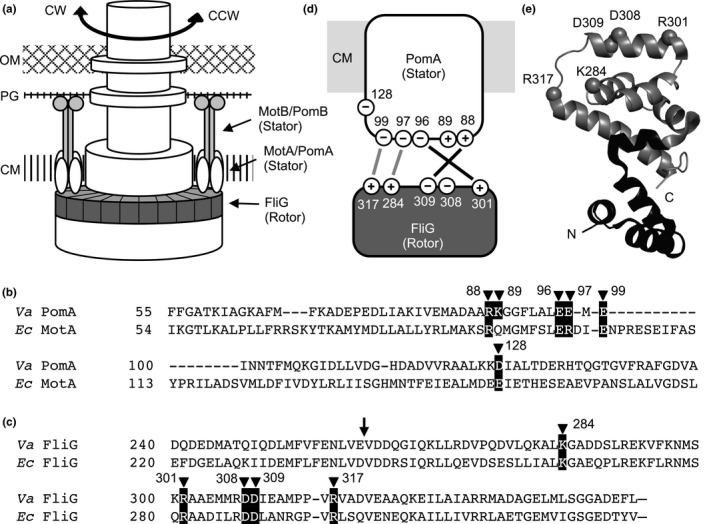
The stator and the rotor of the flagellar motor. (a) The flagellar motor at the bacterial membrane. PomA (MotA), indicated in white, and PomB (MotB), indicated in light gray, form the stator complex. PomA (MotA) interacts with the rotor FliG (indicated in dark gray), which forms the membrane‐proximal part of the C ring, to rotate the flagellum. CM, PG, and OM represent cytoplasmic membrane, peptidoglycan layer, and outer membrane, respectively. (b) Sequence alignment of amino acid residues of PomA (MotA), the component of the stator from *Vibrio alginolyticus* and *Escherichia coli*. Important charged residues for motility are indicated by filled arrowheads and shown in black boxes. The numbering of the amino acid residues on the tops of the sequences is from *V. alginolyticus*. (c) Sequence alignment of amino acid residues of FliG, the component of the rotor from *V. alginolyticus* and *E. coli*. Important charged residues for motility are indicated as described in (b). The position of the junction in the FliG^EV^ chimera is indicated by an arrow at the top of the sequences. (d) Interface of the rotor–stator interaction. Highly conserved charged residues that are important for motility are indicated. The numbering of the amino acid residues is from *V. alginolyticus*. Black lines indicate interactions required for motor function, and gray lines indicate additional interactions reported in *Vibrio*. (e) Structure of the C‐terminal domain of FliG from *Thermotoga maritima* (Protein Data Bank code: 1LKV) (Brown et al., 2002). The corresponding positions of the mutated residues in this study are shown as balls and labeled as the amino acid residues in *V. alginolyticus*. In the chimeric rotor protein FliG^EV^, the portions from *E. coli* and *V. alginolyticus* are shown in black and gray, respectively

The bacterial flagellum is powered by a rotary motor that is driven by an ion‐motive force that turns the flagellar filaments to enable bacteria to swim in liquids (Larsen, Reader, Kort, Tso, & Adler, 1974; Manson, Tedesco, Berg, Harold, & van der Drift, 1977). The core of the rotor is composed of FliG, FliM, and FliN. Those components form a hollow, ring‐like structure, called the C ring, located at the bottom of the flagellar basal body near the inner membrane and facing the cytoplasm (Francis, Sosinsky, Thomas, & Derosier, 1994). FliG is the most important part of the rotor necessary for torque generation; it interacts directly with the stator complex through its C‐terminal domain (Lloyd, Tang, Wang, Billings, & Blair, 1996; Zhou, Lloyd, & Blair, 1998). The stator is embedded in the membrane and is formed by a complex of MotA and MotB for H^+^‐driven motors, or PomA and PomB for Na^+^‐driven motors (Kojima & Blair, 2004; Sato & Homma, 2000). These proteins form an ion channel and couple ion translocation to the interaction of the cytoplasmic domain of the A subunit with the rotor (Figure 1a) (Berg, 2003; Sowa & Berry, 2008). The B subunit is thought to anchor the stator to the peptidoglycan layer, and possibly another stationary component of the flagellum, through its C‐terminal domain (De Mot & Vanderleyden, 1994; Terashima, Fukuoka, Yakushi, Kojima, & Homma, 2006).

On the basis of several lines of experimental evidence, it is thought that the mechanism of flagellar rotation is the same in all bacterial species. First, the essential parts required for the rotor–stator interaction are interchangeable among species. Chimeric FliG proteins, with an N‐terminal domain from *Escherichia coli* and a C‐terminal domain from *Thermotoga maritima* (Lloyd, Whitby, Blair, & Hill, 1999), *Rhodobacter sphaeroides* (Morehouse, Goodfellow, & Sockett, 2005), *Vibrio cholerae* (Gosink & Häse, 2000), or *V. alginolyticus* (Yorimitsu, Mimaki, Yakushi, & Homma, 2003), are functional in *E. coli*. The hybrid stator complex formed by MotA from *R. sphaeroides* and PomB from *V. alginolyticus* functions in *V. alginolyticus* (Asai, Kawagishi, Sockett, & Homma, 1999). The stator complex made from PomA and chimeric PotB (the N‐terminal domain of PomB from *V. alginolyticus* and C‐terminal domain of MotB from *E. coli*), and the stator made from MotA of the extreme thermophilic *Aquifex aeolicus* and chimeric MotB (the N‐terminal domain from *A. aeolicus* and C‐terminal domain from *E. coli*), are functional in *E. coli* (Asai, Yakushi, Kawagishi, & Homma, 2003; Takekawa et al., 2015).

Second, the torque–speed relationship of various flagellar motors is similar. This relationship has been determined for *E. coli* (Chen & Berg, 2000) and *V. alginolyticus* (Sowa, Hotta, Homma, & Ishijima, 2003), and for motors driven by the chimeric stator, PomA/PotB in *E. coli* (Inoue et al., 2008). The absolute values of maximum torque and maximum speed are different for each motor, but the overall trend is the same.

Finally, the functionally important charged residues needed for rotation are highly conserved across bacterial species (Figures 1b,c, and S1). The electrostatic interactions between the charged residues in the C‐terminal domain of FliG and the cytoplasmic domain of MotA (or PomA) are crucial for rotor–stator function (Attmannspacher, Scharf, & Schmitt, 2005; Morimoto, Nakamura, Hiraoka, Namba, & Minamino, 2013; Takekawa, Kojima, & Homma, 2014; Yakushi, Yang, Fukuoka, Homma, & Blair, 2006; Zhou et al., 1998). Specifically, in *E. coli*, R90 (R88 in *V. alginolyticus*) and E98 (E96) in MotA, and R281 (R301), D288 (D308), and D289 (D309) in FliG are the residues of primary importance, whereas E150 (D128) in MotA, and K264 (K284) and R297 (R317) in FliG are of secondary importance (Lloyd & Blair, 1997; Zhou & Blair, 1997). Interactions between R90 (R88) in MotA and D289 (D309) in FliG and between E98 (E96) in MotA and R281 (R301) in FliG are important for motor function (Zhou et al., 1998). Furthermore, these interactions have distinct functions in *Salmonella enterica* (Morimoto et al., 2013). The interaction between R90 (R88) in MotA and D289 (D309) in FliG is critical for stator assembly into the motor, whereas the interaction between E98 (E96) in MotA and R281 (R301) in FliG is important for torque generation. Similarly, in *Sinorhizobium meliloti*, R90 (R88), E98 (E96), and E150 (D128) in MotA, and R294 (R301) and D302 (D309) in FliG are important for motor function. In particular, E150 (D128) is essential for torque generation and R90 (R88) and E98 (E96) are important for controlling the rotary speed (Attmannspacher et al., 2005). In *Bacillus subtilis*, E98 (E96) and E102 (E99) in MotA, and R94 (R88), K95 (K89), and E107 (E99) in MotP (Na^+^‐driven stator) are important for motor function (Takahashi & Ito, 2014). In *V. alginolyticus*, additional charged residues collectively participate in torque generation (Takekawa et al., 2014).

The flagellar motor rotates bidirectionally in most species, and the rotational direction is controlled by the chemotaxis or phototaxis systems (Berry & Armitage, 1999; Blair, 1995). In the chemotaxis signaling pathway, CheY is phosphorylated by the chemoreceptor‐coupled CheA kinase (Hess, Oosawa, Kaplan, & Simon, 1988), and phosphorylated CheY binds to FliM in the C ring (Welch, Oosawa, Aizawa, & Eisenbach, 1993). That binding induces conformational changes that affect protein–protein interactions in the C ring (Paul, Brunstetter, Titen, & Blair, 2011; Sarkar, Paul, & Blair, 2010). Those changes somehow cause a conformational change in the FliG C‐terminal domain that switches the rotational direction from counterclockwise (CCW) to clockwise (CW) (Lam et al., 2012; Lee, Ginsburg, Crovace, Donohoe, & Stock, 2010; Lloyd et al., 1999; Minamino et al., 2011).

This model for the rotational switch implies that, after the rotational switch, the interface of the rotor–stator changes and the contribution of each charged residue that is important for rotation in the CCW direction is different from that in the CW direction. However, most studies have focused on the rotational switch itself (Bai et al., 2010; Wang, Yuan, & Berg, 2014) rather than on the mechanisms of CCW versus CW rotation, although it is known that the flagellar motor rotates stepwise similarly in both directions (Nakamura, Kami‐ike, Yokota, Minamino, & Namba, 2010), and that the overall trend of the torque–speed relationship depends on the rotational direction (Yuan, Fahrner, Turner, & Berg, 2010).

In this study, we used site‐directed mutagenesis to investigate if the functional roles of these conserved charged residues are symmetric (i.e., that their functions are independent of the rotational direction) or asymmetric (i.e., that their functions are dependent on the rotational direction). Our results show that some substitutions of charged residues in the rotor significantly affect rotation in only one direction, and we discuss possible differences in the roles of those residues in the generation of rotation.

## 2 EXPERIMENTAL PROCEDURES

### 2.1 Bacteria, plasmids, and growth media

*E. coli* DFB245 (*motA*, Δ*fliG*), a kind gift from David Blair, was used as a host cell (Zhou et al., 1998). pTY301 and pTY402 (Yorimitsu et al., 2003) were used for expression of the rotor proteins FliG^E^ from *E. coli* and chimeric FliG^EV^, respectively. pJN726 (Hizukuri, Kojima, Yakushi, Kawagishi, & Homma, 2008) and pYS3 (Yakushi et al., 2006) were used for expression of the stator proteins, MotA/MotB and PomA/PotB, respectively. These plasmids were cotransformed into DFB245. Ampicillin and chloramphenicol were used at 50 μg/ml and 25 μg/ml, respectively.

Bacteria were cultured in LB medium, 1% (w/v) Bacto^™^ Tryptone, 0.5% (w/v) yeast extract, and 0.5% (w/v) NaCl, overnight at 37°C from freezer stocks. These cultures were diluted 100‐fold into fresh TB medium, 1% (w/v) Bacto^™^ Tryptone and 0.5% (w/v) NaCl, with 0.02% (w/v) arabinose. After culture for another 4 h at 30°C, these bacteria were used for further analyses.

### 2.2 Tethered‐cell assay

Cultured bacteria were washed three times with Rotation Buffer (10 mM potassium‐phosphate buffer, 0.1 mM EDTA, 10 mM lactic acid, and 100 mM NaCl, pH 7.0). Cells were passed through a needle (26‐gauge) 30 times to shear off flagellar filaments, then washed again. The flow‐chamber, with a volume of 10–20 μl, was made from a cover glass and a glass slide with double‐bonded tape. The anti‐FliC serum (Nishiyama & Kojima, 2012) was diluted 200‐fold, infused into the chamber, and incubated for over 1 h. After washing with 200 μl Rotation Buffer, sheared cells were infused and incubated for ~20 min to allow them to attach to the glass surface. After they were washed with 200 μl Rotation Buffer, the tethered bacterial cells were observed using a phase‐contrast microscope (BH‐2, Olympus) with a 40× objective (A40PL, numerical aperture 0.65, Ph2, Olympus).

We first observed the rotation of bacteria for ~30 s under unstimulated conditions. Then, we infused 50 μl Rotation Buffer with 10% (w/v) glycerol to cause cells to rotate in the CW direction and observed them for ~90 s. Next, we infused 50 μl Rotation Buffer with 10 mM serine and 10% (w/v) glycerol to cause them to rotate in the CCW direction and observed them for another ~90 s. These observations were recorded on a PC using a WV‐1550 CCD camera (National) and PowerDirector^®^ software (CyberLink) at 30 frames/s.

### 2.3 Data analysis

The rotational speeds of the cells were measured by replaying the movies using Move‐tr/2D software (Library Co.). We selected cells that rotated in both directions continuously, counted the number of frames in which the cells rotated for 10 (or 5) rounds, and calculated their rotational speeds. We fitted the CCW–CW speed data to a straight line that passes through the origin. The slope indicates the average ratio of the rotational speed in the CW direction to the rotational speed in the CCW direction.

### 2.4 Multiple sequence alignment

Multiple amino acid sequence alignment was performed using ClustalW. In the case of the alignment of the stator, some sequences around a gap were modified manually.

### 2.5 Swimming on soft‐agar plates

Aliquots of overnight cultures (1 μl) were spotted on TB soft‐agar plates, 1% (w/v) Bacto^™^ Tryptone, 0.5% (w/v) NaCl, and 0.25% (w/v) Bacto Agar^™^, with 0.02% arabinose. The plates were incubated at 30°C for 7 h.

### 2.6 Motile fraction and swimming speed

Cultured bacteria were diluted in TB medium at 100‐fold and observed using a dark‐field microscope (BH‐2, Olympus) with a 10× objective (DPlan 10, numerical aperture 0.25, Olympus). Movies were recorded as described above. The motile fractions and swimming speeds of bacteria were analyzed using Move‐tr/2D software (Library Co.).

## 3 RESULTS

### 3.1 Symmetric rotation in both directions

**Figure 2:**
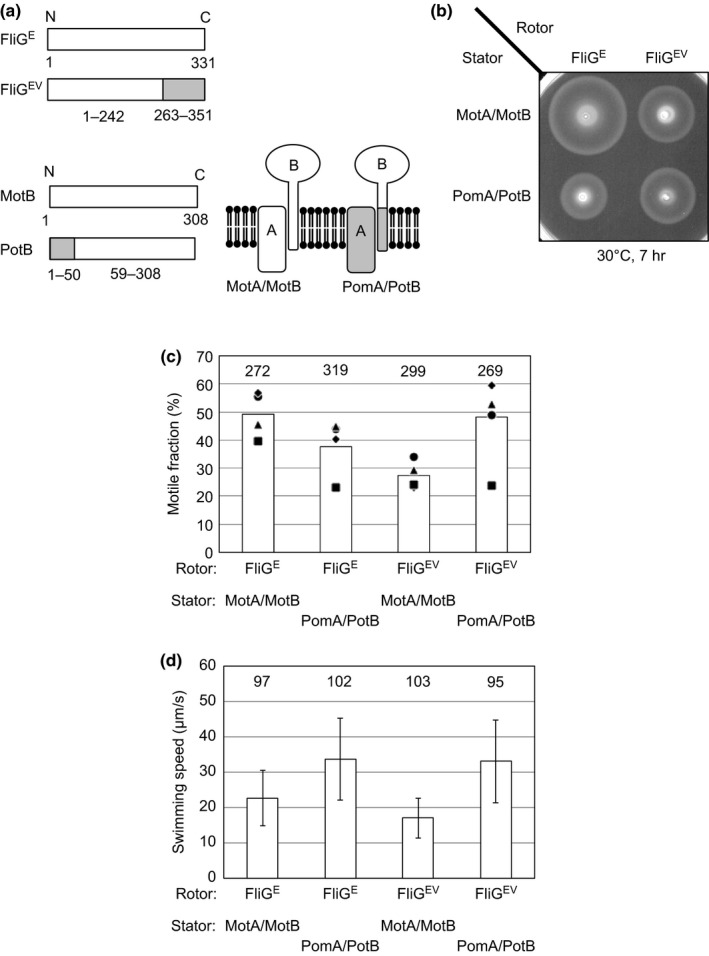
Functional analysis of chimeric rotors and chimeric stators. (a) Schematic representations of chimeric stators and chimeric rotors. The chimeric rotor, FliG^EV^, is composed of the N‐terminal domain of FliG from *Escherichia coli* (amino acids 1–242) and the C‐terminal domain of FliG from *Vibrio alginolyticus* (amino acids 263–351). The chimeric stator, PotB, is composed of the N‐terminal domain of PomB from *V. alginolyticus* (amino acids 1–50) and the C‐terminal domain of MotB from *E. coli* (amino acids 59–308). (b) Motility of chimeric motors on soft‐agar plates. (c) Motile fractions of chimeric motors in solution. Small circles, triangles, squares, and diamonds represent results from four independent experiments. The motile fractions of the combined data from these four experiments are shown as bar graphs. The total number of cells examined in these experiments is indicated at the top of each bar. (d) Swimming speeds of chimeric motors in solution. Average swimming speeds and standard deviation are shown as bar graphs and error bars, respectively. The total number of cells examined from three independent experiments is shown at the top of each bar

Our ultimate goal is to understand the rotational mechanism of the *Vibrio* flagellar motor. Unfortunately, however, it is difficult to observe its rotation directly with traditional methods because *Vibrio* cell has a single polar flagellum at its cell pole (Allen & Baumann, 1971). Therefore, we used *E. coli* cells in which the chimeric proteins expressed because they have multiple flagella around the cell surface, and are suitable for rotational analysis by established approaches such as tethered cell assay. We expressed a chimeric stator and rotor that are functional in *E. coli* and allow *E. coli* cells to swim (Yakushi et al., 2006) (Figure 2). FliG^EV^ is a chimeric FliG composed of the N‐terminal region of *E. coli* FliG and the C‐terminal region of *V. alginolyticus* FliG (Yorimitsu et al., 2003) (Figure 2a). PotB is a chimeric protein that consists of the N‐terminal region of *V. alginolyticus* PomB and the C‐terminal region of *E. coli* MotB (Figure 2a), which can function with *V. alginolyticus* PomA as a Na^+^‐driven stator in *E. coli* (Asai et al., 2003). When these chimeric proteins were coexpressed in *E. coli*, the bacteria spread on soft‐agar plates (Figure 2b). Observing individual cells in a liquid environment under an optical microscope, we found that the fraction of motile cells was similar to that of cells driven by *E. coli* motors, whereas the swimming speed was higher (Figure 2c,d).

**Figure 3:**
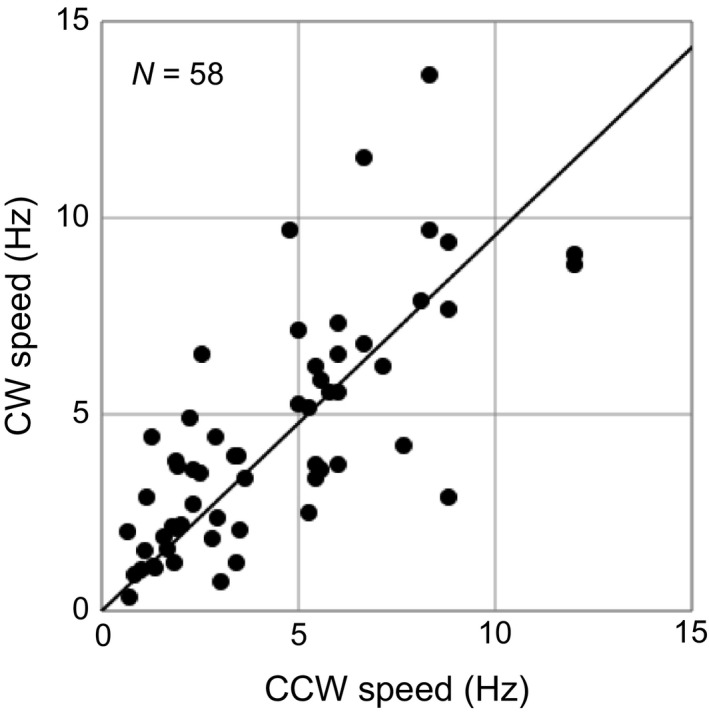
Counterclockwise (CCW)–clockwise (CW) speeds of the chimeric motor, FliG^EV^ and PomA/PotB. Each symbol indicates a cell that rotates in both rotational directions. N indicates the number of bacteria analyzed. The thick line represents the linear fit that passes through the origin. The slope of this line indicates the ratio of the rotational speed in the CW direction to the rotational speed in the CCW direction

To examine the functions of the conserved charged residues in the stator and rotor components of the flagellar motor, we analyzed the rotational dynamics in both directions using a tethered‐cell assay. We used glycerol and serine as a repellent and an attractant, respectively, to control the flagellar rotational direction (Mesibov & Adler, 1972; Oosawa & Imae, 1983). After exchanging buffers in the observation chamber from repellent (10% glycerol) to attractant (10 mM serine), we measured the rotational speeds in the CW and CCW directions for each cell. As shown in Figure 3, the rotational speeds in each direction vary widely because of differences in cell size and the position of the tethering flagellum along the cell cylinder. If the torque is constant in each direction and its rotational frictional drag coefficient is the same in both directions, the plots in the CCW–CW curve must be linear. Thus, we analyzed the ratio of CW speed to CCW speed by fitting those data to a straight line. Variations from this line are presumably due to loose tethering, which allows for different rotational geometries in the two directions during the solution exchange. The correlation coefficient was relatively high, which supported the fitting (Table 1). Moreover, the slope of the fitted line was close to 1 for cells expressing the unmutated chimeric stator and rotor proteins. The equality of rotational speeds in both directions indicates that the flagellar motors produce a similar torque in both directions under high load conditions, such as the tethered‐cell assay. This result is consistent with previous observations of motor function under high load (Yuan et al., 2010) in which rotational speeds over a broad range of loads were measured using variously sized attached beads to flagellar stubs.

**Table 1:** Statistical analysis of rotating cells in both directions

Mutant	#	CCW speed (Hz)		CW speed (Hz)		Fitting		Correlation coefficient
		*Mean*	*SD*	*Mean*	*SD*	Slope	Error	
WT	58	4.33	2.83	4.46	2.95	0.956	0.0522	0.747
PomA (stator)								
R88A	43	4.09	2.33	4.43	2.76	1.05	0.0542	0.802
K89A	43	4.68	2.34	4.23	1.98	0.838	0.0462	0.692
E96Q	37	4.44	3.30	4.61	3.07	0.972	0.0408	0.911
E97Q	35	3.94	1.99	4.04	1.86	0.932	0.0655	0.599
E99Q	45	4.23	2.64	4.24	2.10	0.895	0.0479	0.771
D128N	34	4.42	3.31	4.51	3.13	0.946	0.0537	0.855
AA	36	3.72	3.30	3.38	2.42	0.783	0.0502	0.843
QQQ	44	4.04	3.01	5.00	3.68	1.18	0.0566	0.867
AAQQQ	0	na	na	na	na	na	na	na
E97K	16	2.63	1.51	4.04	2.00	1.24	0.213	0.209
FliG (rotor)								
K284A	49	1.06	1.13	5.48	3.24	3.13	0.387	0.456
R301A	32	4.45	2.32	4.09	2.52	0.893	0.0612	0.733
D308A	32	4.77	2.22	5.10	2.22	1.01	0.0557	0.735
D309A	35	5.13	2.78	4.84	2.65	0.922	0.0356	0.891
R317A	35	1.35	1.19	5.27	3.11	2.74	0.348	0.460
K284E	16	0.846	0.657	4.13	1.88	3.33	0.689	0.203
R317D	22	1.32	1.05	3.15	2.50	1.85	0.328	0.404
PomA (stator)‐FliG (rotor)								
E99Q‐R317A	21	0.954	0.800	5.57	2.26	3.76	0.690	0.229
E97K‐K284E	21	0.580	0.410	3.91	2.48	5.37	0.828	0.413

#, number of bacterial cells analyzed; WT, wild‐type; AA, R88A/K89A double mutant; QQQ, E96Q/E97Q/E99Q triple mutant; AAQQQ, R88A/K89A/ E96Q/E97Q/E99Q quintuple mutant; na, not applicable; CCW, counterclockwise; CW, clockwise.

### 3.2 Mutations in the stator

We introduced charge‐neutralizing mutations into the stator to examine the importance of the charged residues. If the mutations affected the rotational speed, there would be two possible explanations. In the first case, the slope of the CCW–CW speed curve would be close to 1, as with the wild‐type motor. This would mean that the mutation affected rotation in both directions in the same way. In the second case, the slope of the CCW–CW speed curve would not be close to 1. This would mean that the mutation affected the function preferentially in one of the rotational directions.

**Figure 4:**
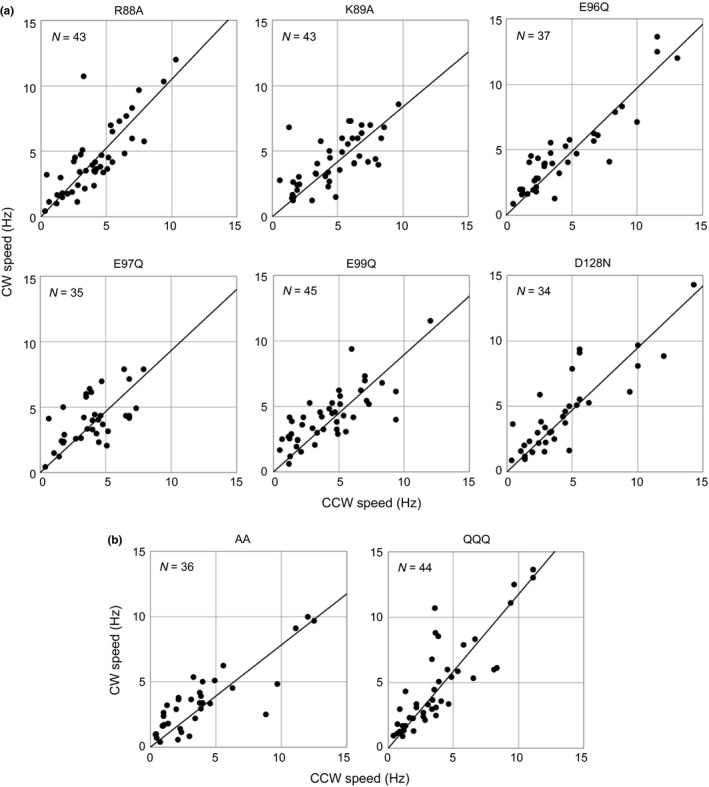
Mutations in charged residues of the stator. (a) Counterclockwise (CCW)–clockwise (CW) speeds of single residue replacement mutants of the stator. (b) CCW–CW speeds of double and triple mutants of the stator, containing 2 or 3 residue replacements, respectively. AA, R88A/K89A double mutant; QQQ, E96Q/E97Q/E99Q triple mutant. Plots were constructed as described in Figure 3

We examined six mutations in A subunit (PomA) of the stator: R88A, K89A, E96Q, E97Q, E99Q, and D128N (Figure 1b,d). The mutational sites are conserved charged residues in *V. alginolyticus*. Although the probability of success of the switching depended on the mutation, cells expressing all of the mutant proteins could rotate continuously in both directions after exchanging the buffer solution with repellent or attractant. The CCW–CW speed results for the stator mutants are shown in Figure 4a. For all six mutants, the slope of the fitted line is almost one, and is similar to that of the wild‐type motor (Table 1). Thus, the motors containing each charge‐neutralized residue substitution function similarly in both rotational directions. In other words, the functions of the charged residues of the stator are symmetric. To distinguish if those mutations affected the rotational function equally in both rotational directions or had no effect, we analyzed the rotational speeds of these bacteria. All of the strains with single charge‐neutralized residue substitutions in the stator rotated at a comparable speed in both directions as cells expressing the original chimera (Table 1). This result suggests that none of the single mutants of the stator affected rotation.

PomA, the stator component from *Vibrio* species, has additional charged residues compared to MotA from *E. coli*; we had previously suggested that these charged residues function redundantly (Takekawa et al., 2014). Therefore, we assessed the function of the double, triple, and quintuple charge‐neutralizing residue substitutions: R88A/K89A (AA), E96Q/E97Q/E99Q (QQQ), and R88A/K89A/E96Q/E97Q/E99Q (AAQQQ). The cells with the quintuple mutation did not rotate in either directions in the tethered‐cell assay (Table 1). For the double and triple mutants, the slope of the CCW–CW speed plot was close to 1 (Figure 4b and Table 1). Additionally, the average rotational speed of the AA mutant was reduced slightly in both rotational directions (Table 1). Taken together, these results confirm that the charged residues are redundant for flagellar rotation and contribute equally to torque generation in both rotational directions.

### 3.3 Mutations in the rotor

**Figure 5:**
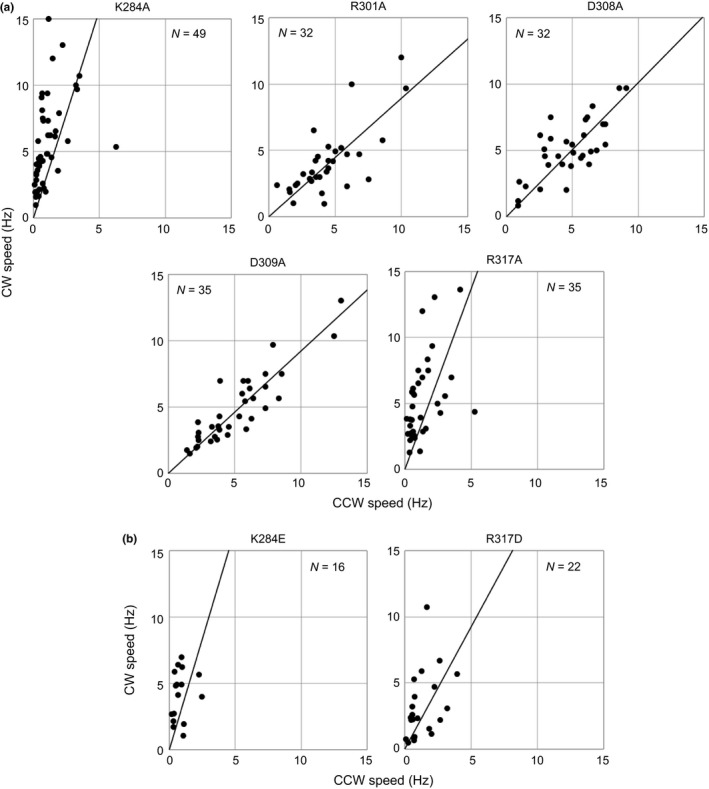
Mutations in conserved charged residues of the rotor. (a) Counterclockwise (CCW)–CW speeds of charge‐neutralizing mutants of the rotor. (b) CCW–CW speeds of charge‐reversing mutants of the rotor. Plots were constructed as described in Figure 3

Next, we examined five charge‐neutralizing residue substitutions in the rotor: K284A, R301A, D308A, D309A, and R317A (Figure 1c,d). The positions of the residue substitutions are conserved charged residues in *V. alginolyticus*. R301, D308, and D309 are located in an alpha helix in the C‐terminal domain of FliG, and K284 and R317 are located beside the helix and close to each other (Figure 1e). For bacteria with 3 of those five mutations in the rotor, the slope of the CCW–CW speed curve was the same as for wild‐type cells (Figure 5a and Table 1). Bacteria with the other two mutations, K284A and R317A, had a steeper slope than the wild‐type cells. The rotational speeds of those two mutants were reduced significantly only in the CCW direction compared to the speeds of the wild‐type strain and other mutants. These results suggest that the K284A and R317A mutations selectively impaired the rotational function in the CCW direction.

We then investigated the effect of charge‐reversing mutations on K284 and R317 of FliG. As determined by tethered‐cell assay, cells expressing the K284E and R317D mutations also exhibited a steeper CCW–CW speed slope and reduced average rotational speed in the CCW direction, but not in the CW direction (Figure 5b and Table 1). Therefore, the positive charge in these residues is essential for rotational function selectively in the CCW direction.

### 3.4 Dual mutations in both the stator and the rotor

**Figure 6:**
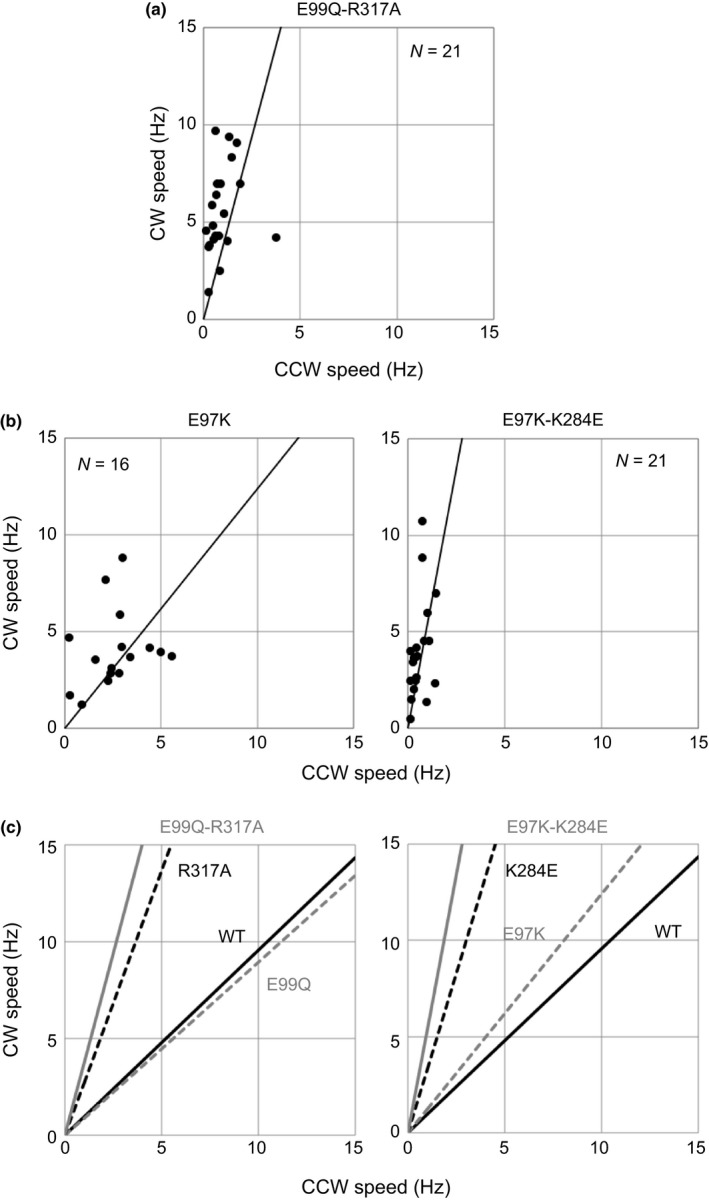
Dual mutation of both the stator and the rotor. (a) Counterclockwise (CCW)–clockwise (CW) speeds of the E99Q‐R317A double mutant. (b) CCW–CW speeds of a dual mutant of E97K‐K284E and a single mutant of E97K. Plots were constructed as described in Figure 3. (c) Summarized CCW–CW speed relationship for cells with dual mutations, E99Q‐R317A (left figure) and E97K‐K284E (right figure). The fitted lines are shown for easy comparisons. The relationships of wild‐type, single mutations in the stator, single mutations in the rotor, and dual mutants in both the stator and the rotor are indicated as a solid black line, a dashed gray line, a dashed black line, and a solid gray line, respectively

We have comprehensively investigated the effects of dual mutations in the stator and the rotor of *Vibrio* on motility, and found several interactions between the stator and the rotor (Takekawa et al., 2013). E99 (in PomA)‐R317 (in FliG) and E97 (in PomA)‐K284 (in FliG) are interactions that are important for proper performance of the *Vibrio* motor. Thus, we tested the possibility that the dual mutants (E99Q‐R317A and E97K‐K284E) restored the asymmetry observed in the CCW–CW speed plots for the single R317A and K284E residue substitutions (Figure 5a,b). In both dual mutants, we observed steep slopes and reduced rotational speeds in the CCW direction in comparison with wild‐type motors (Figure 6a,b and Table 1), as we found for the single mutations in the rotor (Figure 6c).

## 4 DISCUSSION

In this study, we investigated the effects of the charged residues in the rotor and stator components of the flagellar motor, taking into account their effects on rotation in the CCW and CW directions (Figures 4, 5, and Table 1). Those residues are highly conserved and have been suggested to be important for the motility of many bacteria. We found that most single charge‐neutralizing residue substitutions in the stator and the rotor did not affect rotational function and were independent of the rotational direction. Double and quintuple charge‐neutralizing residue substitutions in the stator, AA and AAQQQ, suppressed the rotational function in both rotational directions slightly and completely, respectively. These results indicate that some electrostatic interactions are redundantly involved in the rotation of flagella and those charged residues have a symmetric role in rotation. On the other hand, residue substitutions in two charged residues, K284 and R317, in the rotor selectively impaired CCW rotation, suggesting that they have an asymmetric role in rotation. We measured the rotational speed of the flagellar motor in both directions under high load conditions using the tethered‐cell assay. These charged residues, K284 and R317, in the rotor appear to be important for the rotational function of the flagellar motor in the CCW direction.

In the current model of the rotational switch of the flagellar motor, it is thought that FliG changes its conformation to orient the position of its C‐terminal domain (Lam et al., 2012; Lee et al., 2010; Lloyd et al., 1999; Minamino et al., 2011). This rearrangement changes the interface of the rotor–stator interaction to induce rotary torque in the two opposite directions. The movement of the C‐terminal domain of FliG relative to the base of the C ring is supported by a cross‐linking experiment (Paul, Brunstetter, et al., 2011. We found that residue substitutions in two residues, K284 and R317, of the rotor protein FliG impaired rotational function selectively in the CCW direction. These results strongly suggest that the rotor, FliG, changes the interaction‐site with the stator depending on the rotational direction.

Two residues, K284 and R317, in the rotor have strong asymmetric effects on rotation. Moreover, the interaction of these rotor residues with the stator does not affect their asymmetric function (Figure 6). We interpret that to mean that K284 and R317 do not directly associate with torque generation. K284 of FliG appears in two conformations in the crystal structure of FliG. In one conformation, the side chain of K284 is exposed to the solvent and, in the other conformation, it is less exposed, as previously pointed out by Brown, Hill, and Blair (2002). In the crystal structure of FliG from *T. maritima*, the side chain of K266 (the equivalent of K284 in *V. alginolyticus*) is hydrogen‐bonded to the backbone carbonyl groups of residues L292 and G296 of FliG (Brown et al., 2002; Lloyd et al., 1999). In the structure of the complex of FliG and FliM from *T. maritima*, the side chain of K266 is hydrogen‐bonded to the carbonyl group of L292 but not to that of G296 (Vartanian, Paz, Fortgang, Abramson, & Dahlquist, 2012). We predict that this subtle conformational change occurs during the rotational switch, which is consistent with our data. The side chain of K284A cannot be hydrogen‐bonded to the main chain and alters the conformation required for CCW rotation, resulting in reduced torque generation in the CCW direction. Furthermore, we recently found that CW‐biased rotation of a mutant of FliG, A282T, is caused by an additional hydrogen‐bond to the main chain in the C‐terminal domain of FliG (Miyanoiri et al., 2017). This result also implies that intramolecular interaction in FliG is important for rotational function. In contrast, the side chain of R317 is exposed freely to solvent in the crystal structure. However, substitution of cysteine for this residue induces cross‐linking in the motor, implying that the residues in adjacent subunits can approach close enough to form a disulfide cross‐link (Lowder, Duyvesteyn, & Blair, 2005). Thus, we propose that this residue has a role in stabilizing the conformation of FliG in the C ring of the motor in the CCW direction.

The results of this study raise two interesting questions. Why does neutralization of the charged residues in the stator not have an asymmetric effect on rotation, and why is the CW rotation unaffected by residue substitution of any of the charged residues? Principally, charged residues must be involved in torque generation in both rotational directions, as discussed above. One possibility is that there are other, as yet unidentified, charged residues in the stator and/or the rotor that participate in torque generation. The R232E mutation in the chimeric stator, PomA/PotB, reduces the swimming ability of *E. coli* (Inoue et al., 2008; Yakushi et al., 2006). R232 is located in the cytoplasmic C‐terminal domain of PomA, where there are many charged residues that are important for motor function (Obara, Yakushi, Kojima, & Homma, 2008). Although it is difficult to interpret the role of the charge of a histidine residue in protein–protein interactions because its pK_a_ value is near neutral pH, the residue substitution H136Y of PomA from *V. alginolyticus* impairs its rotational function (Fukuoka, Yakushi, & Homma, 2004; Takekawa et al., 2013). These residues might be candidates for charged residues that directly participate in torque generation.

Another possibility is that electrostatic interactions play a minor role in torque generation, especially in the CW direction. The motor from *R. sphaeroides* alternates between rotation in the CCW direction and cessation of rotation, even though its stator and rotor retain all of the highly conserved charged residues (Pilizota et al., 2009). On the other hand, *S. meliloti* has right‐handed rather than left‐handed helical flagellar filaments that rotate only CW (Götz & Schmitt, 1987), even though they change speed. One group reported that the motor of *Caulobacter crescentus* generates more torque in CCW rotation than in CW rotation (Lele, Roland, Shrivastava, Chen, & Howard, 2016). Interestingly, these three bacteria belong to Alphaproteobacteria. Therefore, despite the conservation of the charged residues across a wide range of species, the exact mechanisms that support CCW and CW rotation remain to be determined; they must differ among species and be related to their evolutionary histories.

## CONFLICT OF INTEREST

None declared.
