# Supplementary material for: The role of conserved charged residues in the bidirectional rotation of the bacterial flagellar motor
Source: Microbiologyopen. 2018 Mar 24;7(4):e00587. doi: 10.1002/mbo3.587 (PMC6079164; doi:10.1002/mbo3.587)
Supplement: Supplementary file 1 [file MBO3-7-e00587-s001.pdf]

Supporting Information

The role of conserved charged residues in bidirectional rotation of the bacterial flagellar motor

Yasuhiro Onoue, Norihiro Takekawa, Tatsuro Nishikino, Seiji Kojima and Michio Homma

Figure Legends

Figure S1. Amino acid sequences of the rotor and the stator of flagellar motors. (A) Multiple sequence alignment of amino acid residues of PomA (MotA), the component of the stator. Important charged residues for motility are indicated by filled arrowheads and are highlighted in red. Numbering of amino acid residues at the top of the sequences is from *Vibrio alginolyticus*. (B) Multiple sequence alignment of amino acid residues of FliG, the component of the rotor. Important charged residues for motility are indicated by filled arrowheads and are highlighted in red. Numbering of amino acid residues at the top of the sequences is from *V. alginolyticus*. The position of the junction in the $FliG^{EV}$ chimera is indicated by an arrow at the top of the sequences. *Aa*, *Aquifex aeolicus*; *Bs*, *Bacillus subtilis*; *Ec*, *Escherichia coli*; *Rs*, *Rhodobacter sphaeroides*; *Se*, *Salmonella enterica*; *Sm*, *Sinorhizobium meliloti*; *Tm*, *Thermotoga maritima*; *Va*, *Vibrio alginolyticus*; *Vc*, *Vibrio cholerae*.

A

```
                                                                 88▼▼89  96▼▼97 ▼99
Va PomA      1  ---MDLATLLGLIGGFAFVIMAMVLGGSIG---MFVDVTSILIVVGGSIFVVLMKFTMGQ
Rs MotA      1  ---MDIAAAIGLIGAIVMVVGSMIYAGGVA---PFVDIPSLVIVVAGTAFIVLAMKPLPV
Bs MotP      1  MKRFDYLTPVGFVLGTIIIVIGIISGSGVSGFRSFLDLTSFFIVTGGLCAAVFISFPPSE
Aa MotA      1  ---MDVGTIIGIIAAFLLILISILIGGSIT---AFINVPSIFIVVGGGMAAAMGAFPLKD
Bs MotA      1  ---MDKTSLIGIIILAFVALSVGMVLKGVSFS--ALANPAAILIIIAGTISAVVIAFPTKE
Ec MotA      1  -----MLILLGYLVVLGTVFGGYLMTGGSLG--ALYQPAELVIIAGAGIGSFIVGNNGKA
Se MotA      1  -----MLILLGYLVVIGTVFGGYVMTGGHLG--ALYQPAELVIIGGAGIGAFIVGNNGKA
Sm MotA      1  -----MNIIIGLLVTFGCILGGYMAMGGHLE--VLNQPFELMIIGGAGIGGFIMANSMKV
                            :* :     :  . :  .         : :   :.*:  ..     .

Va PomA     55  FFGATKIAGKAFM---FKADEPEDLIAKIVEMADAARKGGFLALEE-M-E----------
Rs MotA     55  FLGHFKAMMKVFK---PSRFDMNEVISTMVELSNLARKDGIMALEGKA------------
Bs MotP     61  LKKAPSVLKQAFI---RQEDNVKDLVKTFVSLSDHARKHGLLSLDDQARE----------
Aa MotA     55  FIRGVLAIKKAFL---WKPPDLNDVIETIGEIASKVRKEGILALEGDI-ELY--------
Bs MotA     56  IKKVPTLFRVLFKE--NKQLTIEELIPMFSEWAQLARREGLLALEASI-ED---------
Ec MotA     54  IKGTLKALPLLFRRSKYTKAMYMDLLALLYRLMAKSRQMGMFSLERDI-ENPRESEIFAS
Se MotA     54  IKGTMKAIPLLFRRSKYTKSMYMDLLALLYRLMAKSRQQGMFSLERDI-ENPKESEIFAS
Sm MotA     54  VKDTGKALGEAFRHKVPKERЕYLDTLGVLYSLMRDLRTKSRNEIESHI-DNPEESSIFQS
                .          *          : :  :         *   .   ::

                                                    ▼128
Va PomA    100  --------INNTFMQKGIDLLVDG-HDADVVRAALKKDIALTDERHTQGTGVFRAFGDVA
Rs MotA    100  --------VPDAFFEKGLQLLVDG-TDEAKLVKQLKYEIKAMKARHEAYQGAVKAWIDIG
Bs MotP    108  --------IKDPFLKKGLLLAIDG-WDEETIRLVMDSEIAAMEERHRKGRRVFEKAGEFA
Aa MotA    103  -------YQKDPLLGDMIRMLVDG-IDINDIKATAEMALAQLDEKMSTEVAVWEKLADLF
Bs MotA    104  --------VDDAFLKNGLSMAVDG-QSAEFIRDIMTEEVEAMEDRHQAGAAIFTQAGTYA
Ec MotA    113  YPRILADSVMLDFIVDYLRLIISGHMNTFEIEALMDEEIETHESEAEVPANSLALVGDSL
Se MotA    113  YPRILADAVMLDFIVDYLRLIISGNMNTFEIEALMDEEIETHESEAEVPANSLAMVGDSL
Sm MotA    113  APTVLQNKELTAFICDYVRLIIIGNARSHEIEALMDEEIQTITHDKMKCYHAMTTMGDAL
                           :: . : : : *      :        :
```

B

```
Va FliG     180  IANLEEVQPSALAELNEIMEKQFAGQAGAQAAKIGGLKAAAEIMNYLDNNVEGLLMEQIR
Vc FliG     177  IANLEEVQPSALAELNEIMEKQFAGQAGAQAAKIGGLKAAADIMNYLDNNIESVLMEGMR
Ec FliG     161  IATFGGVQPAALAELTEVLNGLLDGQN-LKRSKMGGVRTAAEIINLMKTQQEEAVITAVR
Se FliG     161  IATFGGVQPAALAELTEVLNGLLDGQN-LKRSKMGGVRTAAEIINLMKTQQEEAVITAVR
Rs FliG     168  IASLNTVQPEALADLQQVMQRKFKASTTMRASQIGGVKAAARIMNFTRTATEARILKDIR
Bs FliG     165  IAVMDRTSPEIINEVERILEQKLSSAFTQDYTQTGGIEAVVEVLNGVDRGTEKTILDSLE
Tm FliG     162  IALLERTSPEVVKEIERNLEKKISGFVSRTFSKVGGIDTAAEIMNNLDRTTEKKIMDKLV
Sm FliG     175  AVNIKNVNAKAAAIIEARVIEIIEEMETERNS--PGPAKIAEVMNELEKPQVDTLLASLE
                  . :  ...    :   :   :          :    *    . ::*         ::  :

                                          ↓                         ▼284
Va FliG     240  DQDEDMATQIQDLMFVFENLVEVDDQGIQKLLRDVPQDVLQKALKGADDSLREKVFKNMS
Vc FliG     237  EKDEDLATQIQDLMFVFENLVEVDDQGIQKLLRDVPQDVLQKALKGADDTLREKIFKNMS
Ec FliG     220  EFDGELAQKIIDEMFLFENLVDVDDRSIQRLLQEVDSESLLIALKGAEQPLREKFLRNMS
Se FliG     220  EFDGELAQKIIDEMFLFENLVDVDDRSIQRLLQEVDSESLLIALKGAEPPLREKFLRNMS
Rs FliG     228  KDDKDLMQAIQDNMFVFDNLIKSDDRSLQTLLRAVDNETLVLALKGADEGLRAKILGCMS
Bs FliG     225  IQDPDLAEEIKKRMFVFEDIVTLDNRAIQRVIRDVENDDLLLSLKVASEEVKEIVFNNMS
Tm FliG     222  QENPELADEIRRRMFVFEDILKLDDRSIQLVLREVDTRDLALALKGASDELKEKIFKNMS
Sm FliG     233  TISTDSAKKVRPKIFLFDDILFMPQRSRVQLFNDVSADVITMALRGSAAELRESILASIG
                   . :    :   :*:*::::   ::.   ::. *    :  :*: :   ::  .:  :.

              301▼      308▼▼309     ▼317
Va FliG     300  KRAAEMMRDDIEAMPP-VRVADVEAAQKEILAIARRMADAGELMLSGGADEFL--
Vc FliG     297  KRAAEMMKDDLEAMPP-IKVSDVEAAQKEILSIARRMADNGEIMLGGGADEFL--
Ec FliG     280  QRAADILRDDLANRGP-VRLSQVENEQKAILLIVRRLAETGEMVIGSGEDTYV--
Se FliG     280  QRAADILRDDLANRGP-VRLSQVENEQKAILLIVRRLAETGEMVIGSGEDTYV--
Rs FliG     288  TRAAATVRDEMEALGP-VRLTDVQAAQKQIIAVARQMSDEGTIVLAGRGGEQMV-
Bs FliG     285  QRMVETFKEEMEFMGP-VRLKDVEEAQSRIVSIVRKLEEAGEIVIARGGGDDIIV
Tm FliG     282  KRAAALLKDELEYMGP-VRLKDVEEAQQKIINIIRRLEEAGEIVIARGGGEELIM
Sm FliG     293  ARQRRMIESDLAAGDAGINPRDIAIARRSITQEAIRLSASGQLELKEKEPEAA--
                  *    ...::    . :.  ::   :   *     ::    * : :
```

Fig. S1
